# VEGF Contributes to Mesenchymal Stem Cell-Mediated Reversion of Nor1-Dependent Hypertrophy in iPS Cell-Derived Cardiomyocytes

**DOI:** 10.1155/2021/8888575

**Published:** 2021-04-10

**Authors:** Denise Philipp, Michelle Holthaus, Vida Basoah, Kurt Pfannkuche, Laura Suhr, Thorsten Wahlers, Adnana Paunel-Görgülü

**Affiliations:** ^1^Department of Cardiothoracic Surgery, Heart Center of the University of Cologne, Cologne, Germany; ^2^Center for Physiology and Pathophysiology, Institute for Neurophysiology, University of Cologne, Medical Faculty, Cologne, Germany

## Abstract

Myocardial hypertrophy is present in many heart diseases, representing a strong predictor of adverse cardiovascular outcomes. Regarding therapeutic intervention, mesenchymal stem cells (MSCs) have been suggested to significantly reduce cardiac hypertrophy and progression to heart failure. Preconditioning of MSCs was previously demonstrated to highly improve their paracrine activity resulting in modulation of immune responses and the progression of diseases. Here, we studied the effects of bone marrow-derived preconditioned MSCs on hypertrophied induced pluripotent stem cell-derived cardiomyocytes (iPS-CM) and also sought to identify MSC-derived antihypertrophic molecules. Phenylephrine (PE) was used to induce hypertrophy in murine iPS-CM, and markers of hypertrophy were identified by microarray analysis. Murine MSCs were treated with IFN-*γ* and IL-1*β* to enhance their paracrine activity, and transcriptional profiling was performed by microarray analysis. Hypertrophied iPS-CM were subsequently cocultured with preconditioned MSCs or MSC-conditioned medium (CM), respectively. Effects on hypertrophied iPS-CM were studied by cell area quantification, real-time PCR, and western blot. In some experiments, cells were incubated with fractions of MSC-CM obtained by ultrafiltration or by MSC-CM supplemented with inhibitory antibodies. Intracellular and extracellular levels of vascular endothelial growth factor (VEGF) were evaluated by western blot and ELISA. PE-induced hypertrophy in iPS-CM was associated with an upregulation of neuron-derived orphan receptor (Nor1) expression, activation of Akt, and inhibition of both strongly prevented hypertrophy induction in iPS-CM. VEGF secreted by preconditioned MSCs provoked hypertrophy regression in iPS-CM, and a negative correlation between *Nor1* expression and hypertrophic growth could be evidenced. Our results demonstrate that *Nor1* expression strongly supports hypertrophy in iPS-CM. Moreover, the secretome of preconditioned MSCs triggered regression of hypertrophy in iPS-CM in a VEGF-dependent manner. We suggest that the delivery of the MSC-derived secretome may represent a therapeutic strategy to limit cardiac hypertrophy. However, additional *in vivo* studies are needed to prove this hypothesis.

## 1. Introduction

Left ventricular hypertrophy (LVH) is a common manifestation of many forms of cardiac disease, including myocardial infarction and hypertension [1]. It is widely recognized as a major risk factor for the development of heart failure and cardiac sudden death representing an established predictor of cardiovascular morbidity and mortality. LVH is a complex and multifactorial condition involving a process of adaptive remodeling, which is usually a compensatory mechanism in response to increased hemodynamic load [2]. Although called compensatory, prolonged hypertrophy increases the risk for progression into heart failure (decompensated hypertrophy). LVH is accompanied by various cardiac and molecular changes, including increase in protein synthesis, cell sizes, and inflammatory and fibrogenic responses which lead to myocardial remodeling and subsequent ventricular dysfunction [1]. Inflammation was shown to be a prominent hallmark of LVH in animal [3] and human studies [4]. Increased wall stress associated with LV overload promotes remodeling processes characterized by increased proinflammatory cytokine expression, leukocyte infiltration, and myocardial destruction. Since a variety of signaling pathways have been shown to be involved in the progression of cardiac hypertrophy [5], understanding the mechanisms underlying the development of cardiac hypertrophy is essential for the treatment of cardiovascular disease. Accordingly, reversal of myocardial hypertrophy has been considered a therapeutic strategy for the reduction of adverse remodeling and prevention of heart failure [6].

The NR4A orphan nuclear receptor subfamily consists of the highly homologous receptors Nur77 (NR4A1), Nurr1 (NR4A2), and Nor1 (NR4A3) which can be regulated by various stimuli, including growth factors, hormones, and inflammatory signals. Recently, there has been much attention paid to the function of these receptors in the cardiovascular system [7]. Nor1 was shown to regulate a number of essential biological processes including inflammation [8], and the first evidence has already been provided for its prohypertrophic properties [9, 10]. Therapeutic strategies targeting hypertrophic markers to dampen the inflammatory response may hold promise for beneficial intervention in LVH. In this context, modulation of a macrophage phenotype has been proposed as a new therapeutic approach, as these cells are recognized as key players in cardiovascular diseases. Exacerbated local inflammation in the myocardium mediated by proinflammatory microRNA-155-expressing M1 macrophages induced cardiac hypertrophy and failure in response to pressure overload [11]. In turn, microRNA-155 knockout in macrophages, but not in cardiomyocytes, markedly reduced inflammation, hypertrophy, and dysfunction in pressure overload. Recently, regression of cardiac hypertrophy by stem cell transplantation and subsequent macrophage polarization towards an anti-inflammatory M2 phenotype was demonstrated [12]. Besides, stem cell-based therapies are currently considered for the treatment of heart failure. Several studies, including clinical studies, have shown that the transplantation of mesenchymal stem cells (MSCs) may enhance tissue perfusion and angiogenesis and preserve or regenerate the myocardial tissue [13–16]. In this context, the prior research of our group has demonstrated that transfer of bone marrow-derived MSCs to the myocardium improves global function and remodeling in mice [17]. MSCs are able to secrete cytokines, growth factors, microRNAs, and exosomes which are potent for improving neovascular formation, attenuating inflammation and adverse remodeling, and inducing endogenous cardiac regeneration. Rather, the secretome of bone marrow-derived MSCs was found to favor an immunosuppressive environment [18–20]. At present, MSCs are still the most suitable for clinical treatment [21]. Preconditioning of MSCs highly increases their efficacy *in vitro* and *in vivo*, thus preventing loss of biological function, enhancing their biological activity, and representing a more attractive strategy for clinical use [22].

In this study, we have established an *in vitro* model of cardiac hypertrophy on the basis of murine iPS cell-derived cardiomyocytes (iPS-CM) and studied the hypertrophic response. We further investigated whether preconditioned MSCs might reverse hypertrophy in iPS-CM and identified VEGF as a major contributor of MSC-mediated hypertrophy regression.

## 2. Materials and Methods

### 2.1. Cell Culture Conditions for Murine iPS-CM and Hypertrophy Induction

Differentiated, nonproliferative murine iPS-CM were kindly provided by Dr. Kurt Pfannkuche (Institute of Neurophysiology, University of Cologne). These cells exhibit spontaneous contraction and unimpaired physiological function as evidenced by action potential generation, functional hormonal regulation, and sensitivity to ion channel blockers [23]. Cells were seeded on fibronectin-coated culture dishes (2.6 *μ*g/cm^2^) and cultured in IMDM medium with GlutaMAX (Gibco), 20% FCS (PAN), 1% nonessential amino acids (Gibco), 0.1 mM 2-mercaptoethanol (Gibco), and 100 U/ml penicillin/streptomycin (Sigma-Aldrich). The medium was renewed every day. To induce hypertrophy, cells were starved in IMDM medium with GlutaMAX supplemented with 1% FCS for 24 h and further treated with L-phenylephrine (PE, 100 *μ*M, Sigma-Aldrich) for additional 24 h. In some experiments, hypertrophied iPS-CM were cultured in medium supplemented with MSC-conditioned medium (CM) or fractions from MSC-CM and PE (100 *μ*M) in the presence or absence of polyclonal goat anti-mouse VEGF_164_ antibodies (R&D Systems).

### 2.2. Isolation, Characterization, and Activation of Bone Marrow-Derived MSCs

Isolation and characterization of mouse bone marrow-derived MSCs were previously reported by our group in detail. Cells were positive for classical MSC markers including Sca-1, CD49e, CD44, and CD29 and did not express CD45 and CD11b, respectively. The MSC phenotype was confirmed by the analysis of its differentiation potential into chondrocytes, adipocytes, and osteoblasts [20]. For experiments, MSCs were stimulated with 30 ng/ml recombinant murine IFN-*γ* (PeproTech) and 3 ng/ml recombinant murine IL-1*β* (PeproTech) in MSC culture medium (Pan-Biotech) supplemented with 2.5 ng/ml human basic fibroblast growth factor FGF (FGF-b, PeproTech), 100 U/ml penicillin, and 10 *μ*g/ml streptomycin (Sigma-Aldrich) for 24 h. Preconditioned MSCs possess anti-inflammatory and immunomodulatory capacities [20] and were used for coculture experiments.

To gain MSC-CM, preconditioned MSCs were further cultured in serum-free IMDM medium with stable glutamine (Gibco) for additional 24 h. Then, supernatants were collected and stored at -80°C until further processing. To prove if MSCs retain their immunomodulatory properties upon serum starvation, nitrite levels in culture supernatants were quantified using a modified Griess reagent (Sigma-Aldrich) as already described [20]. Supernatants containing high levels of nitrite, indicating upregulated *iNOS* expression, were used for experiments.

### 2.3. Coculture Conditions

For coculture experiments, hypertrophied iPS-CM were indirectly cocultured with preconditioned MSCs (5 : 1 ratio) in IMDM medium supplemented with 1% FCS and 100 *μ*M PE for 24 h. For this, conditioned MSCs were placed in transwell inserts with 0.4 *μ*m pore size (Corning) to avoid direct cellular interactions.

### 2.4. Immunofluorescence Analysis

iPS-CM (1.5 × 10^4^) were grown on fibronectin-coated coverslips in a 12-well plate, starved for 24 h, and treated with 100 *μ*M PE in the presence of 1% FCS. Then, cells were cocultured with preconditioned MSCs or treated with MSC-CM or fractionated supernatant, respectively, in the presence of PE. After 24 h, cells were fixed with 4% paraformaldehyde for 20 min and permeabilized with 0.1% Triton X-100, and nonspecific binding sites were blocked using 1% BSA diluted in PBS. Cells were further incubated with Alexa Fluor® 555 phalloidin (Thermo Scientific) in blocking solution for 20 min to stain F-actin, followed by counterstaining with DAPI. The coverslips were then washed and mounted on glass slides. Fluorescent images were obtained using a Nikon Eclipse Ti-U inverted microscope (Eclipse Ti-U 100, Nikon, Germany) using the software NIS-Elements version 3.00. Cell surface areas (*μ*m^2^) of 100 or more cells per condition were measured in 10 randomly chosen fields.

### 2.5. Real-Time PCR

Total RNA from iPS-CM was isolated by using the RNeasy Mini Kit (Qiagen) and reverse transcribed with High Capacity cDNA Reverse Transcription Kit (Applied Biosystems). Target genes were amplified by real-time PCR using StepOnePlus or QuantStudio 3 Real-Time PCR System (Applied Biosystems) and Power SYBR Green PCR master mix (Applied Biosystems). The following *Nor1* primers were used: for: 5′-AGACGCCGAAACCGATGT-3′ and rev: 5′-TCGGACAAGGGCATTCA-3′. Gene expression was normalized to 18S rRNA. All samples were run in triplicate. Fold expression was calculated using the 2^−ΔΔ^C_T_ method.

### 2.6. Microarray Analysis

RNA was extracted using RNeasy Mini Kit (Qiagen), and contaminating DNA was removed by DNA-free Kit (Ambion). Gene expression profiling of hypertrophied iPS-CM and preconditioned MSCs was performed using GeneChip Mouse Genome 430.2 (Affymetrix) or Clariom S Assays (Thermo Fisher) at the Gene Expression Affymetrix Facility headed by Prof. Agapios Sachinidis (Center of Molecular Medicine, Cologne). RNA from 3-5 independent experiments was used.

### 2.7. siRNA Transfection

To knockdown *Nor1* expression, iPS-CM were seeded in 6-well plates or alternatively on coated coverslips in 12-well plates and cultured in IMDM medium+1% FCS. Transfection has been performed using OptiMEM medium (Gibco) and Lipofectamine RNAiMAX Reagent (Invitrogen) according to the manufacturer's instructions. Cells were transfected with 5.5 nM Silencer Select Nor1 siRNA and Silencer Select negative control siRNA (Ambion), respectively, and incubated for 24 h at 37°C and 5% CO_2_. Then, 100 *μ*M PE was added and cells were cultured for additional 24 h. Transfection efficiency was verified by real-time PCR. Cells cultured on coverslips were used for immunofluorescent analysis.

### 2.8. Western Blot

Cells were lysed in RIPA buffer supplemented with protease and phosphatase inhibitor cocktail (Cell Signaling Technology) and sonicated, and protein concentration was quantified by using Pierce BCA Protein Assay Kit (Thermo Fisher Scientific). Protein mixture was separated by SDS-PAGE and transferred to a nitrocellulose membrane (0.2 *μ*m). Membranes were blocked with blocking buffer and further incubated overnight at 4°C with the following antibodies: rabbit anti-mouse phospho-Akt (Ser473), NF-*κ*B p65, pNF-*κ*B p65 (Ser536), HIF-1*α* (Cell Signaling Technology), polyclonal rabbit anti-mouse Nor1 (Abcam), and polyclonal goat anti-mouse VEGF_164_ antibody (R&D Systems). After incubation with a HRP-conjugated goat anti-rabbit (Dako) or horse anti-goat (Vector Labs) secondary antibody membranes were developed with UptiLight HRP Blot Chemiluminescent ECL Substrate (Uptima). To normalize protein expression, blots were stripped and reprobed for GAPDH or *β*-actin and Akt, respectively, using a mouse anti-GAPDH antibody (Novus Biological) and anti-mouse *β*-actin and rabbit anti-mouse Akt antibody (Cell Signaling Technology).

### 2.9. ELISA

The levels of VEGF were quantified by ELISA using mouse DuoSet kits according to the manufacturer's instructions (R&D Systems).

### 2.10. Ultrafiltration

Ultrafiltration units with various cutoffs (Vivaspin 6-20, Sartorius, Germany) were first rinsed by centrifuging pure water through the membrane. The medium to be analyzed was then centrifuged at 6000 × *g* until the supernatant reached a tenth of its initial volume, and both the concentrated supernatant and filtrate fractions were collected in plastic tubes and used for experiments.

### 2.11. Statistical Analysis

Data were analyzed with GraphPad Prism 5 software. Experimental data are presented as means with standard error of the mean (SEM). Unpaired data of two groups were analyzed using the unpaired *t*-test. The one-sample *t*-test was used when samples were compared with the reference control sample (set as 1). Normally distributed unpaired data of multiple groups were analyzed using one-way ANOVA with the Newman-Keuls post hoc test. A *p* value less that 0.05 was considered statistically significant.

## 3. Results

### 3.1. Identification of Hypertrophy Markers in PE-Stimulated iPS-CM

**Figure 1:**
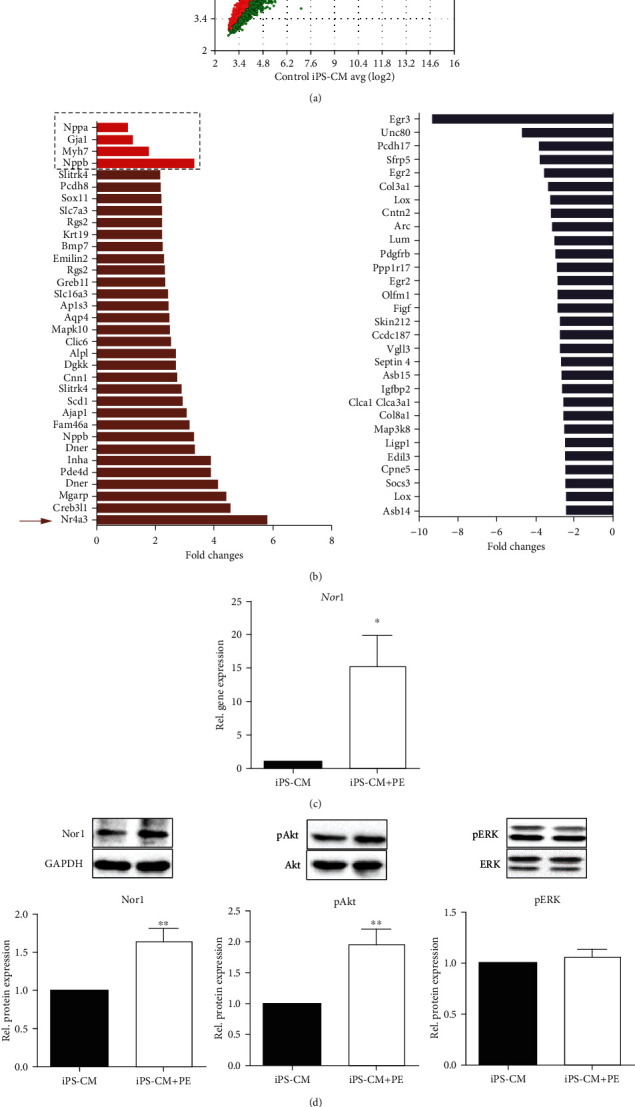
Characterization of hypertrophied iPS-CM. iPS-CM were cultured in the presence of 100 *μ*M phenylephrine (PE) to induce hypertrophy. (a) Scatter plot of microarray data showing log2 of the gene transcripts between iPS-CM and iPS-CM treated with PE. (b) Top 30 up- and downregulated genes in PE-treated iPS-CM. Additional significantly upregulated genes involved in hypertrophic cell growth are highlighted by the dotted square. (c) *Nor1* gene expression was verified by real-time PCR. *n* = 5. (d) Western blot analysis of Nor1 (*n* = 8), normalized to the expression levels of GAPDH. The expression of phosphorylated Akt (pAkt, *n* = 8) and phosphorylated Erk (pERK, *n* = 4) in hypertrophied iPS-CM was normalized to that of the unphosphorylated forms. (e) iPS-CM were seeded on fibronectin-coated coverslips and treated with 100 *μ*M PE for 24 h. Cells were further fixed and incubated with Alexa Fluor 555 phalloidin to stain intracellular F-actin (red). Nuclei were counterstained with DAPI (blue), and the cell area was quantified microscopically. Bar: 100 *μ*m; *n* = 7. ^∗^*p* < 0.05, ^∗∗^*p* < 0.01.

Cardiac hypertrophy is manifested by numerous transcriptional, biochemical, and structural transformations including increased cell size, enhanced protein synthesis, and remodeling of the actin cytoskeleton. However, an *in vitro* hypertrophy model based on murine iPS-CM has not been described so far. Thus, we first started to identify gene markers in iPS-CM regulated by the hypertrophic substance PE. By performing microarray analysis, we found 154 genes to be regulated (fold change in expression >2 or <-2) by PE (Table 1, Figure 1(a)). Among the 46 upregulated genes, the gene *Nr4a3*, encoding for neuron-derived orphan receptor 1 (Nor1), was found to be the most upregulated gene (~6-fold induction) in PE-treated iPS-CM. Upregulation of additional genes involved in hypertrophic cell growth, such as *Nppb* (BNP), *Myh7* (*β*-MHC), *Gja1* (Cx43), and *Nppa* (ANP) [24], was lower when compared to *Nor1/Nr4a3* expression levels (Figure 1(b)). On the basis of these results, we decided to use *Nor1* as a valuable marker of hypertrophic cell growth in iPS-CM. The upregulation by PE was confirmed by real-time PCR (Figure 1(c)). In addition, Nor1 protein was significantly increased in hypertrophied iPS-CM, and increased activity of the kinase Akt, but not Erk, could be detected (Figure 1(d)). Nor1 expression showed a positive correlation with the cell surface area of iPS-CM evidenced by actin staining and immunofluorescence (Figure 1(e)).

### 3.2. Nor1 Expression and Akt Activation Are Mandatory for PE-Induced Hypertrophy in iPS-CM

**Figure 2:**
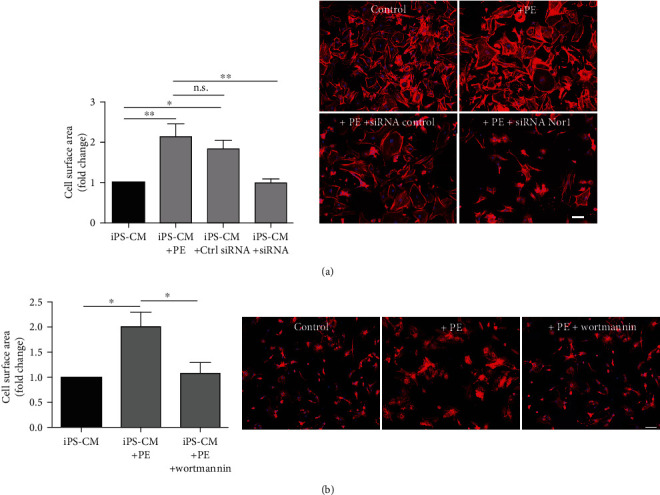
Suppression of Nor1 and Akt activity prevents hypertrophy induction in iPS-CM. (a) iPS-CM were seeded on fibronectin-coated coverslips and transfected with Nor1 siRNA or control siRNA, respectively. After 24 h, cells were further treated with PE (100 *μ*M) to induce hypertrophy. iPS-CM cell size was quantified by histological staining of F-actin (red). *n* = 4. (b) iPS-CM were treated with wortmannin (400 nM) before hypertrophy induction by PE. Cell size was quantified by staining of the actin cytoskeleton. Representative images are depicted. Bar: 100 *μ*m. *n* = 4. n.s. = not significant; ^∗^*p* < 0.05, ^∗∗^*p* < 0.01.

To prove if *Nor1* expression and Akt activation are important for hypertrophic cell growth, we suppressed *Nor1* expression and Akt activity by using gene-specific siRNA or the pI3 kinase inhibitor wortmannin, respectively (Figure S1a-b), before treatment of cells with PE. No hypertrophic cell growth could be observed in the absence of Nor1 (Figure 2(a)) as well as in the case of suppressed Akt activity (Figure 2(b)) indicating that both *Nor1* and activation of Akt are required for hypertrophy induction in iPS-CM. No effect on cell survival was observed (data not shown).

### 3.3. The Secretome of MSCs Induces Hypertrophy Regression in PE-Treated iPS-CM

**Figure 3:**
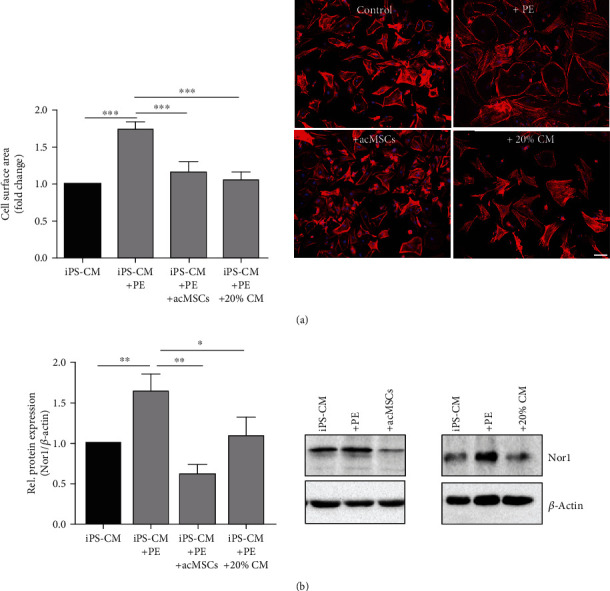
MSC-derived soluble mediators induce hypertrophy regression in iPS-CM. Bone marrow-derived MSCs were stimulated with 30 ng/ml IFN-*γ* and 3 ng/ml IL-1*β* for 24 h. Then, preconditioned MSCs (acMSCS) were indirectly cocultured with hypertrophied iPS-CM. In some experiments, hypertrophied iPS-CM were cultured in medium supplemented with 20% MSC-conditioned medium (CM). (a) Cell area quantification in iPS-CM was performed by immunofluorescent staining. Representative images are displayed. Bar: 100 *μ*m. *n* = 6. (b) Nor1 protein expression in iPS-CM. *n* = 5. ^∗^*p* < 0.05, ^∗∗^*p* < 0.01, and ^∗∗∗^*p* < 0.001.

Stimulation of bone marrow-derived MSCs with IFN-*γ* and IL-1*β* was accompanied by increased activation of NF-*κ*B as well as visible, but not significant, increase in intracellular HIF-1*α* levels (Figure S2a-b). As NF-*κ*B represents the main transcription factor controlling *iNOS* expression in response to IL-1*β* stimulation, nitrite levels were quantified in culture supernatants to validate preconditioning of MSCs [20]. Activation of NF-*κ*B usually results in the upregulation of genes involved in apoptosis, inflammation, and cellular adhesion, among others [25]. To further find out if preconditioned MSCs (acMSCs) influence hypertrophy in iPS-CM, PE-treated iPS-CM were cocultured with preconditioned MSCs by using a transwell system that prevents direct cell-cell interactions. The cell area of iPS-CM was significantly reduced upon coculture with preconditioned MSCs, and similar results were obtained after medium supplementation with 20% MSC-conditioned medium (Figure 3(a)). Of note, the observed effects of the MSC secretome on hypertrophied cells were found to be dose-dependent as medium supplementation with 10% conditioned medium or less did not result in hypertrophy regression (Figure S3). These results indicate that hypertrophy regression is mediated by MSC-derived soluble factors. Notably, hypertrophy regression was associated with downregulation of Nor1 protein expression in iPS-CM (Figure 3(b)).

**Figure 4:**
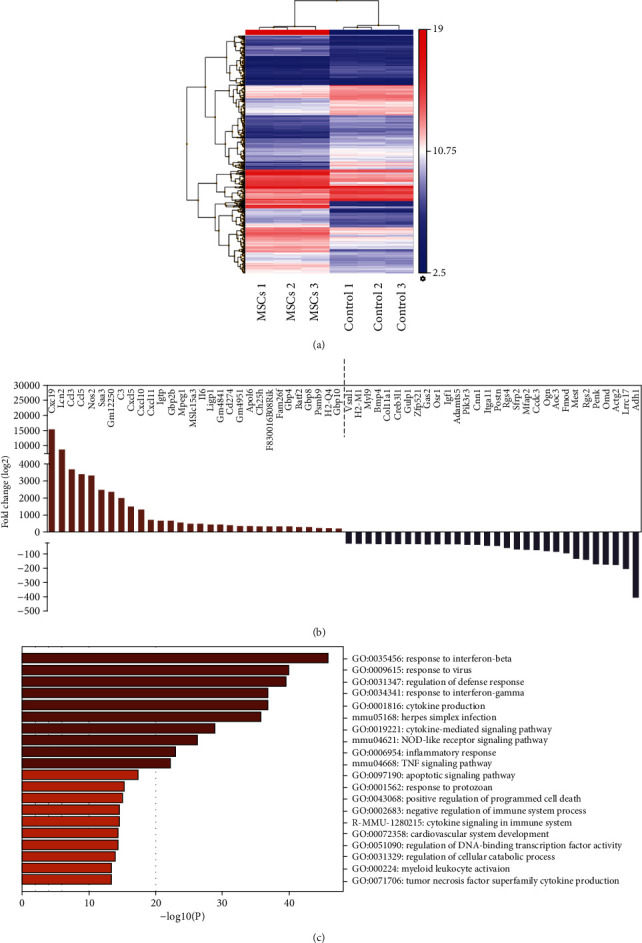
Microarray analysis highlighting differentially expressed genes in preconditioned MSCs. (a) Heatmap representing gene expression in MSCs with and without preconditioning. (b) Top 30 of up- and downregulated genes in MSCs after preconditioning. (c) GO enrichment analysis for upregulated genes in preconditioned MSCs.

We next aimed to identify MSC-derived molecules involved in the regression of hypertrophy. For this, we performed microarray analysis to enclose genes regulated by IFN-*γ* and IL-1*β*. A total of 2026 differentially regulated genes were identified; among them, 1037 were upregulated and 989 were downregulated (Figures 4(a)–4(c)). The top 100 list of significantly regulated genes (>2-fold; <-2-fold change) is given in Table S1. In addition to many genes encoding for chemokines, *iNOS* and *IL-6* were ranked in the top 30 upregulated genes (Figure 4(b)), supporting MSC activation and expression of immunomodulatory factors. Gene ontology (GO) enrichment analyzed by Metascape was used to categorize the upregulated gene networks in preconditioned MSCs (Figure 4(c)). The results suggest that the identified genes are largely involved in cellular responses to interferon-beta, interferon-gamma, cytokine production, and cytokine-mediated signaling pathways. In addition, some of the genes were involved in the regulation of the apoptotic pathway, regulation of DNA-binding transcription factor activity, and myeloid leukocyte activation.

**Figure 5:**
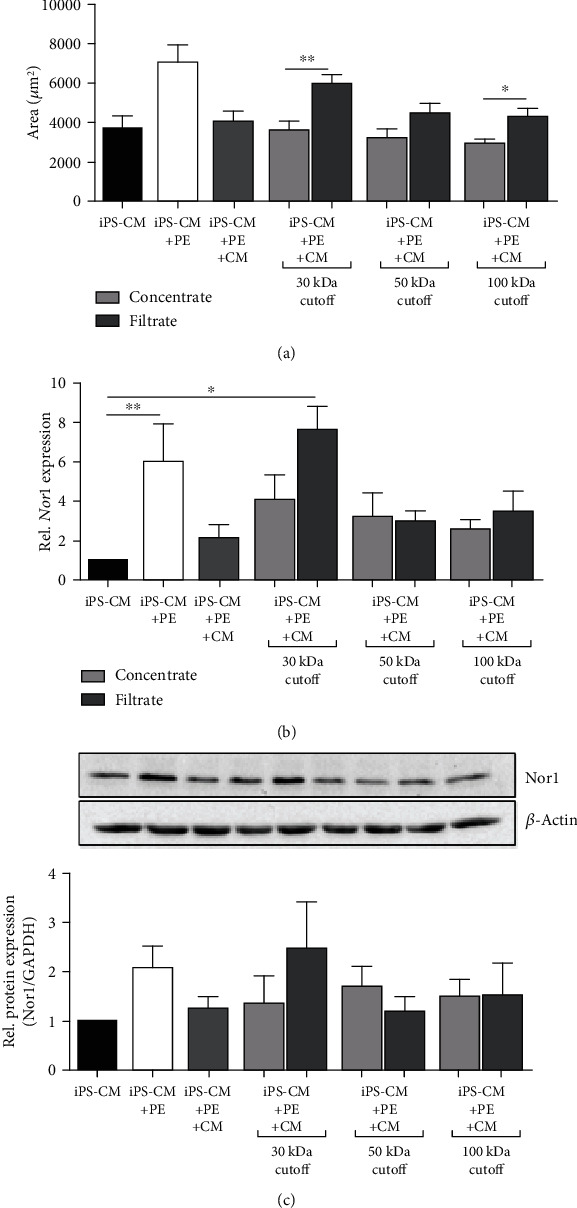
Hypertrophy regression in iPS-CM is mediated by soluble factors larger than 30 kDa. Culture supernatant from preconditioned MSCs was fractionated by ultrafiltration using columns with 30 kDa, 50 kDa, and 100 kDa cutoff. Hypertrophied iPS-CM were treated with the different concentrates and the corresponding filtrates for 24 h. MSC-conditioned medium (CM) was used as a control. (a) Quantification of the cell surface area by immunofluorescence. *n* = 10. Nor1 gene (b) and protein (c) expression was quantified in iPS-CM. *n* = 5. ^∗^*p* < 0.05, ^∗∗^*p* < 0.01.

To further identify MSC-derived molecules which are potentially involved in hypertrophy regression, the supernatant of preconditioned MSCs was concentrated and fractionated by using Vivaspin centrifugal devices with a molecular mass cutoff of 30 kDa, 50 kDa, and 100 kDa. Both the concentrates and the filtrates, containing small molecules, were used to explore their effects on hypertrophied iPS-CM. Histological analysis revealed that all concentrates used for experiments significantly reversed hypertrophy in iPS-CM. In turn, protective effects in cells treated with filtrates were visibly reduced (Figure 5(a)). *Nor1* expression in iPS-CM showed a good correlation with the cell area when cells were treated with the fraction containing molecules < 30 kDa (30 kDa filtrate) and the corresponding concentrate, whereby differences in protein expression were not significantly altered (Figure 4(b)).

### 3.4. VEGF Secreted by Preconditioned MSCs Reverses Hypertrophy in PE-Treated iPS-CM and Downregulates Nor1 Gene Expression

**Figure 6:**
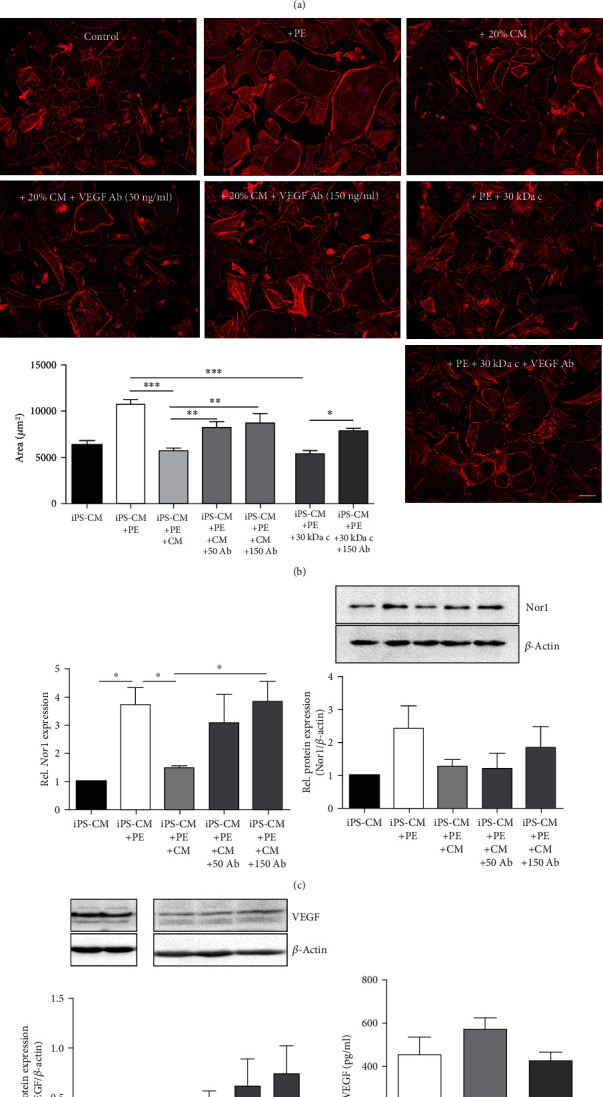
MSC-derived VEGF is essential for hypertrophy regression in iPS-CM. (a) VEGF was quantified in culture supernatants of MSCs stimulated with 30 ng/ml IFN-*γ* and 3 ng/ml IL-1*β* for 24 h and those cultured for additional 24 h in serum-free medium (48 h). In addition, VEGF levels in fractionated MSC-conditioned medium (concentrates and filtrates) were determined by ELISA. *n* = 5. (b) PE-treated hypertrophied iPS-CM were cultured in the presence of 20% MSC-conditioned medium (CM) for 24 h. As a control, cells were cultured in medium supplemented with >30 kDa concentrates. For VEGF neutralization, 50 ng/ml or 150 ng/ml neutralizing anti-VEGF antibodies was added to the culture medium. The cell surface area was quantified by F-actin staining. Representative images are displayed. Bar: 100 *μ*m. *n* = 5. (c) Nor1 gene and protein expression was quantified in iPS-CM cultured in the presence of MSC-CM supplemented with VEGF-neutralizing antibodies. *n* = 4. (d) Intracellular VEGF levels were quantified in hypertrophied iPS-CM cultured in the presence of 20% CM for 24 h. MSCs with and without preconditioning served as a control. *n* = 4. (e) VEGF levels were quantified in culture supernatants of PE-treated iPS-CM cultured in medium supplemented with 20% CM for 24 h. *n* = 6. ^∗^*p* < 0.05, ^∗∗^*p* < 0.01, and ^∗∗∗^*p* < 0.001.

VEGF secreted by bone marrow-derived MSCs was recently reported to reverse hypertrophic cell growth [26, 27]. Having found that molecules involved in hypertrophy reduction seem to be larger than 30 kDa, we next determined if preconditioned MSCs represent a potential source for VEGF. Significant 5.8-fold upregulation of *Vegfa* expression after MSC preconditioning could be detected by microarray analysis (data not shown). VEGF levels were visibly elevated in the supernatants of preconditioned MSCs 24 h as well as 48 h after cytokine stimulation. Moreover, VEGF was detected in all ultrafiltration concentrates (Figure 6(a)) which were previously found to induce hypertrophy regression in iPS-CM (Figure 5(a)). The antihypertrophic effects of MSC-conditioned medium as well as of the medium fraction containing molecules > 30 kDa were strongly mitigated after VEGF inactivation by VEGF-neutralizing antibodies (Figure 6(b)). VEGF neutralization in MSC-conditioned medium increased *Nor1* expression in iPS-CM, although significant increase in Nor1 protein levels could not be detected (Figure 6(c)).

As these results do not exclude the possibility that VEGF may derive from the hypertrophied iPS-CM themselves, we further tested if VEGF expression in iPS-CM becomes regulated by the MSC-conditioned medium. As shown in Figure 6(d), intracellular VEGF protein could be detected in both MSCs and iPS-CM; however, no effect of MSC-conditioned medium on VEGF expression in iPS-CM could be observed. Additionally, neither PE treatment nor culture in the presence of MSC-conditioned medium provoked an increase in VEGF secretion by iPS-CM (Figure 6(e)), suggesting that preconditioned MSCs represent the main source for VEGF.

## 4. Discussion

In this study, we demonstrate for the first time that Nor1 represents an essential molecule for hypertrophy induction in iPS-CM. Further, our data indicate that among others, VEGF secreted by preconditioned murine bone marrow-derived MSCs is effective in reducing hypertrophy in iPS-CM. VEGF-mediated hypertrophy regression strongly correlated with a reduction in *Nor1* gene expression. Thus, delivery of MSC-conditioned medium as well as intrinsic suppression of *Nor1* expression might represent suitable, cell-free strategies to reverse cardiac hypertrophy.

In this study, we established an *in vitro* model of cardiac hypertrophy on the basis of murine iPS-CM. Stimulation of cells with the hypertrophic substance PE increased cell size and induced significant upregulation of the nuclear receptor *Nor1*. However, upregulation of other key hypertrophy genes like *Nppa* (atrial natriuretic factor, ANF), *Nppb* (brain natriuretic peptide, BNP), *Gja1* (connexin 43), and *Myh7* (*β*-myosin heavy chain) was much weaker, indicating that *Nor1* represents the most feasible hypertrophy marker in iPS-CM. Several reports suggest that Nor1 is involved in the pathophysiology of cardiac hypertrophy and heart failure, although very little is known about the underlying molecular mechanisms. Knockdown of Nor1 has been reported to prevent isoprenaline-induced hypertrophy in rat cardiomyocytes, whereas Nor1 overexpression exacerbated cardiac hypertrophy *in vitro* and *in vivo* [9, 10]. In this regard, it is assumed that Nor1 upregulates the expression of hypertrophy markers and additionally promotes PARP-1 activation, resulting in functional impairment and progression of cardiac hypertrophy and heart failure [9]. Although we did not investigate the mechanism by which Nor1 regulates hypertrophy in iPS-CM, we found that silencing of *Nor1* expression by siRNA completely inhibited hypertrophy induction in a similar way as the pI3K inhibitor wortmannin. Thus, *Nor1* expression as well as pI3K/Akt activation is fundamental for hypertrophic cell growth although the involvement of additional factors could be expected.

In the past decade, MSCs have been recognized to represent one of the most promising cellular therapies for cardiovascular diseases due to their ability to self-renew in a long-term manner and the capacity to differentiate into diverse specialized cell types. Additionally, there is a large body of evidence showing that MSCs secrete a variety of biologically active molecules such as growth factors, chemokines, and cytokines [28].

Preconditioning of MSCs greatly improves their proliferative, secretory, and migratory abilities and their abilities to differentiate [22]. Here, we present data demonstrating that preconditioned bone marrow-derived MSCs as well as MSC-conditioned medium promote hypertrophy regression in iPS-CM indicating that this effect is solely dependent on soluble mediators. Importantly, initial experiments demonstrated that MSCs without preconditioning do not lead to hypertrophy regression in iPS-CM after indirect coculture. MSC preconditioning by IFN-*γ* and IL-1*β* led to significant upregulation of NF-*κ*B and consequently induced upregulation of over 1000 genes, most of them encoding for chemokines and cytokines. Hence, both IL-1*β* and IFN-*γ* were reported to promote NF-*κ*B-dependent accumulation of HIF-1*α* under normoxic conditions [29, 30]. As we further found that the molecular weight of these molecules seems to be larger than 30 kDa, we further concluded that most of the upregulated chemokines (molecular weight ~10 kDa) can be excluded as mediators of the protective effects. In fact, many factors such as CCL-2, CCL-3, and CCL-5, whose gene expression was highly increased in preconditioned MSCs, were reported to be upregulated in patients with structural remodeling including hypertrophy and congestive heart disease [31]. Moreover, it has previously been found by Jung et al. that HIF-1*α* is involved in the regulation of VEGF via a pathway dependent on NF-*κ*B [29].

The role of VEGF in cardiac hypertrophy is controversially discussed. On the one hand, VEGF was demonstrated to drive cardiac hypertrophy [32] whereby, on the other hand, VEGF-mediated hypertrophy regression was largely attributed to its ability to increase cardiac angiogenesis [33, 34]. Although MSCs produce remarkable amounts of VEGF [26, 35, 36], VEGF secretion was significantly upregulated by MSC preconditioning. All concentrates of MSC-conditioned medium containing VEGF reversed hypertrophy in iPS-CM, indicating that VEGF induces hypertrophy regression. However, it should be noted that VEGF was identified in all concentrates although concentrators with different cutoffs were used in this study. These results might be explained by the absorbance of the protein to the membrane or the plastic surface of the device. On the basis of the generated results, we cannot exclude that additional MSC-derived soluble molecules may exert antihypertrophic effects by synergistic or additive interactions with VEGF.

VEGF neutralization attenuated the protective effect of MSC-conditioned medium, and upregulation of *Nor1* could be detected at the same time. Additionally, our data do not support the findings of Cai et al. who described a synergistic increase in extracellular VEGF after coculturing hypertrophied cardiomyocytes and bone marrow-derived MSCs [26]. Neither intracellular VEGF upregulation in iPS-CM nor increased VEGF secretion was detected after treatment of cells with MSC-conditioned medium or after indirect coculture, respectively. These observations suggest that preconditioned MSCs seem to represent the main source for VEGF which in turn reverses hypertrophy by suppressing *Nor1* expression in cardiomyocytes.

In this regard, beside contributing to the formation of new vessels, VEGF has additionally been described to induce M1 macrophages to shift to an M2 phenotype [37] and to attenuate cardiac hypertrophy [26, 38]. Thus, the beneficial effects of VEGF *in vivo* are expected to be manifold including direct antihypertrophic effects on cardiomyocytes, macrophage transpolarization into an anti-inflammatory phenotype [37], and angiogenesis [33]. In the light of these results, cytokine preconditioning should be considered a tool to increase the therapeutic efficacy of bone marrow-derived MSCs.

However, there are some limitations in the present study that need to be addressed. First, our study was limited by the use of adult bone marrow-derived MSCs with reduced self-renewal and differentiation capacity, high variations between different batches, and progressive cell senescence [39–41]. Second, the secretome of MSCs also appears to vary significantly, depending on the age of the host, health status, and niches where the cells reside. Besides, NF-*κ*B was previously reported to be critical for the secretion of cytokines and growth factors by MSCs, and several studies proposed that genetic modification of MSCs may improve NK-*κ*B signaling and their paracrine activities [42, 43]. Although we provided evidence for NF-*κ*B activation in preconditioned MSCs, the onset of NF-*κ*B activation may markedly differ between different MSC preparations. Further, we did not consider the complexity of the MSC secretome including microvesicles, microRNAs, exosomes, and mitochondrial transfer among others [44]. The limitations may be largely omitted by the development of more attractive strategies on the basis of iPS cell-derived MSCs. These cells were demonstrated to have higher proliferative capacity, higher telomerase activity, and lower cell senescence than bone marrow-derived MSCs allowing generation of highly homogenous populations without loss of functional quality [39, 45].

Altogether, the results of our study indicate that conditioned medium from IFN-*γ*- and IL-1*β*-stimulated MSCs may represent an appropriate therapeutic approach for the treatment of LVH. Nevertheless, further studies are needed to characterize preconditioned iPS cell-derived MSCs and to evaluate the efficacy and safety of secretome components for the treatment of cardiac hypertrophy *in vivo*.

## 5. Conclusions

Preconditioning of bone marrow-derived MSCs with IL-1*β* and IFN-*γ* strongly improves their paracrine actions. Moreover, VEGF secreted by MSCs reverses Nor1-dependent hypertrophy in iPS-CM. Altogether, delivery of MSC-conditioned medium may represent a promising cell-free therapeutic approach for the treatment of LVH although *in vivo* efficacy requires further investigation.

## Figures and Tables

**Table 1:** Differential gene expression in PE-treated iPS-CM.

Gene symbol	Gene ID	PE 100 *μ*M avg (log2)	Control avg (log2)	Fold change
Nr4a3	1438796_at	7.93	5.4	5.78
Creb3l1	1419295_at	8.49	6.31	4.53
Mgarp	1420774_a_at	7.01	4.87	4.39
Dner	1456379_x_at	5.75	3.72	4.11
Pde4d	1435280_at	8.62	6.67	3.87
Inha	1422728_at	9.91	7.97	3.86
Dner	1423671_at	6.84	5.11	3.31
Nppb	1450791_at	9.94	8.22	3.3
Fam46a	1437868_at	9.58	7.93	3.14
Ajap1	1456197_x_at	5.08	3.48	3.04
Scd1	1415964_at	10.95	9.42	2.9
Slitrk4	1440516_at	7.52	6.01	2.85
Cnn1	1417917_at	10.04	8.6	2.72
Dgkk	1442865_at	7.08	5.68	2.66
Alpl	1423611_at	7.99	6.58	2.65
Clic6	1454866_s_at	6.12	4.8	2.5
Mapk10	1437195_x_at	6.56	5.26	2.46
Aqp4	1425382_a_at	8.83	7.54	2.45
Ap1s3	1455735_at	6.36	5.08	2.42
Slc16a3	1449005_at	7.03	5.75	2.41
Greb1l	1439341_at	5.66	4.44	2.32
Rgs2	1419248_at	8.42	7.22	2.3
Emilin2	1435264_at	12	10.82	2.27
Bmp7	1418910_at	7.54	6.39	2.22
Krt19	1417156_at	5.43	4.28	2.22
Rgs2	1447830_s_at	7.06	5.92	2.21
Slc7a3	1417022_at	9.55	8.42	2.19
Sox11	1453125_at	6.79	5.67	2.17
Pcdh8	1447825_x_at	4.64	3.54	2.15
Slitrk4	1437744_at	9.71	8.61	2.13
Fndc5	1453135_at	6.77	5.68	2.12
Rgs2	1419247_at	8.66	7.59	2.1
Pcdh8	1417051_at	4.44	3.38	2.08
Bmp7	1432410_a_at	6.67	5.62	2.07
Syt7	1460081_at	8.3	7.26	2.06
Synm	1457275_at	7.98	6.93	2.06
Gp1bb	1422977_at	6.73	5.69	2.06
Bdh1	1452257_at	9.86	8.83	2.04
Pmaip1	1418203_at	8.49	7.46	2.04
Sox11	1429372_at	6.23	5.2	2.04
Kdelr3	1418538_at	8.77	7.75	2.04
Fkbp11	1417267_s_at	6.51	5.49	2.04
Olfml2b	1423915_at	10.71	9.68	2.03
Aqp4	1434449_at	10.01	8.99	2.03
Sprr1a	1449133_at	7.54	6.53	2.02
Synpo	1427045_at	8.17	7.16	2.02
Ddit4l	1451751_at	6.23	7.23	-2
Maob	1434354_at	7.26	8.26	-2
Mmp2	1416136_at	5.97	6.97	-2
Sema5a	1434776_at	6.18	7.18	-2
Phkg1	1422315_x_at	6.46	7.46	-2.01
Kcnd2	1422834_at	6.62	7.62	-2.01
Ccdc187	1430052_at	5.45	6.46	-2.01
Fmod	1437718_x_at	4.98	5.98	-2.01
Shisa9	1435424_x_at	3.79	4.8	-2.01
Cdo1	1448842_at	9.23	10.24	-2.01
Phkg1	1425164_a_at	6.8	7.81	-2.01
Epb41l2	1459619_at	5.89	6.91	-2.01
Scn7a	1436043_at	8.07	9.08	-2.02
Fbln2	1423407_a_at	6.62	7.63	-2.02
Fmod	1456084_x_at	6.16	7.17	-2.02
Clec2l	1433764_at	7.95	8.97	-2.02
Prss35	1434195_at	4.2	5.22	-2.03
	1445148_at	7.17	8.19	-2.03
	1446742_at	3.72	4.74	-2.03
	1439174_at	3.82	4.84	-2.03
Tuba8	1419518_at	6.29	7.32	-2.04
	1440565_at	5.79	6.81	-2.04
Atp1a2	1443823_s_at	10.16	11.19	-2.04
Parp8	1442157_at	4.55	5.58	-2.04
	1457338_at	5.27	6.3	-2.04
Scn7a	1427495_at	5.07	6.1	-2.05
4833420G17Rik	1419637_s_at	7.81	8.85	-2.05
Rgs12	1429380_at	6.68	7.71	-2.05
Rgs4	1416286_at	5.76	6.81	-2.07
Socs2	1446085_at	4.48	5.53	-2.07
Dlk1	1449939_s_at	8.7	9.75	-2.07
Kcnj3	1444025_at	7.85	8.9	-2.07
	1445659_at	4.08	5.14	-2.07
Ctla2a, Ctla2b	1416811_s_at	4.16	5.22	-2.08
Tm4sf1	1450958_at	5.6	6.66	-2.08
Ssbp2	1429951_at	6.91	7.97	-2.09
	1442609_at	5.47	6.53	-2.09
Gucy1a3	1434141_at	9.83	10.89	-2.09
Cdh11	1450757_at	8.41	9.47	-2.09
Ptn	1448254_at	8.92	9.99	-2.1
Col8a1	1455627_at	6.76	7.83	-2.1
Cdh11	1435120_at	4.22	5.29	-2.1
Socs3	1455899_x_at	7.92	8.99	-2.11
Dpm1	1459961_a_at	6.05	7.14	-2.12
Angpt1	1421441_at	5.37	6.46	-2.13
Col18a1	1426955_at	8.21	9.3	-2.13
Pcdh17	1436920_at	7.42	8.51	-2.14
Figf	1438953_at	5.95	7.05	-2.14
Atp1a2	1434893_at	8.27	9.37	-2.15
Cyr61	1457823_at	9.73	10.84	-2.15
Atp1a2	1427465_at	9.12	10.23	-2.15
Kcnip2	1436275_at	5.85	6.96	-2.16
Ano3	1443612_at	3.87	5	-2.18
Batf3	1453076_at	4.8	5.92	-2.18
Hmga2	1422851_at	5.9	7.03	-2.19
Figf	1449528_at	6.74	7.88	-2.19
	1443551_at	7.19	8.32	-2.19
Bgn	1448323_a_at	6.99	8.13	-2.2
Adamts5	1422561_at	6.06	7.2	-2.2
Scn7a	1436044_at	8	9.14	-2.21
Olfm1	1425784_a_at	7.02	8.16	-2.21
Rufy3	1442786_s_at	3.92	5.08	-2.23
Cyr61	1442340_x_at	10.04	11.21	-2.24
Gucy1a3	1420534_at	7.06	8.22	-2.24
Junb	1415899_at	9.02	10.19	-2.25
Zfp36	1452519_a_at	8.69	9.87	-2.26
Fbln5	1416164_at	6.08	7.25	-2.26
Kctd12	1434881_s_at	6.48	7.66	-2.27
Cdc14a	1436913_at	4.4	5.59	-2.28
Boc	1426869_at	5.77	6.98	-2.31
Fam155a	1435138_at	5.08	6.29	-2.31
Frem1	1455280_at	5.83	7.07	-2.36
04. Sep	1448729_a_at	7.54	8.78	-2.36
Ramp1	1431413_at	6.08	7.32	-2.37
Bgn	1416405_at	7.65	8.89	-2.37
Asb14	1449547_at	4.13	5.38	-2.38
Lox	1448228_at	7.07	8.34	-2.41
Socs3	1456212_x_at	7.34	8.61	-2.42
	1442813_at	4.08	5.36	-2.42
	1439535_at	7.43	8.71	-2.42
Cpne5	1442166_at	9.85	11.13	-2.42
Edil3	1433474_at	4.25	5.52	-2.43
Iigp1	1419043_a_at	6.02	7.3	-2.43
Map3k8	1419208_at	6.58	7.89	-2.48
Col8a1	1418440_at	5.65	6.99	-2.53
Clca1; Clca3a1	1417852_x_at	5.53	6.87	-2.53
Igfbp2	1454159_a_at	5.81	7.18	-2.59
Asb15	1439836_at	3.86	5.24	-2.61
04. Sep	1455422_x_at	6.53	7.94	-2.65
Vgll3	1453593_at	4.36	5.78	-2.68
Ccdc187	1439194_at	5.83	7.26	-2.68
Skiv2l2	1447517_at	4.13	5.56	-2.69
	1445655_at	5.35	6.83	-2.79
Figf	1438954_x_at	5.54	7.03	-2.81
Olfm1	1455796_x_at	3.97	5.47	-2.81
Egr2	1427683_at	5.5	6.99	-2.82
Ppp1r17	1449240_at	5.16	6.66	-2.84
Pdgfrb	1436970_a_at	4.57	6.12	-2.93
Lum	1423607_at	6.4	7.98	-2.99
Arc	1418687_at	5.94	7.57	-3.1
Cntn2	1435165_at	6.09	7.75	-3.16
Lox	1416121_at	7.48	9.15	-3.19
Col3a1	1427884_at	5.98	7.71	-3.32
Egr2	1427682_a_at	5.44	7.26	-3.52
Sfrp5	1436075_at	6.34	8.24	-3.75
Pcdh17	1453070_at	6.1	8.01	-3.77
Unc80	1434989_at	5.54	7.75	-4.64
Egr3	1436329_at	3.84	7.06	-9.27

## Data Availability

The datasets supporting the conclusions of this article are available from the corresponding author upon reasonable request.

## Supplementary Materials

Supplementary Materials Supplementary Figure S1: supplementary figure showing suppression of *Nor1* expression and inhibition of Akt activity by siRNA transfection and wortmannin treatment, respectively. Supplementary Figure S2: supplementary figure showing NF-*κ*B activation and HIF-1*α* upregulation in preconditioned MSCs. Supplementary Figure S3: supplementary figure showing hypertrophy regression in iPS-CM after incubation with different concentrations of MSC-conditioned medium. Supplementary Table S1: supplementary table showing the top 100 of up- and downregulated genes in preconditioned MSCs determined by microarray analysis.
