# Supplementary material for: VEGF Contributes to Mesenchymal Stem Cell-Mediated Reversion of Nor1-Dependent Hypertrophy in iPS Cell-Derived Cardiomyocytes
Source: Stem Cells Int. 2021 Apr 10;2021:8888575. doi: 10.1155/2021/8888575 (PMC8053052; doi:10.1155/2021/8888575)
Supplement: Supplementary Materials — Supplementary Figure S1: supplementary figure showing suppression of Nor1 expression and inhibition of Akt activity by siRNA transfection and wortmannin treatment, respectively. Supplementary Figure S2: supplementary figure showing NF-κB activation and HIF-1α upregulation in preconditioned MSCs. Supplementary Figure S3: supplementary figure showing hypertrophy regression in iPS-CM after incubation with different concentrations of MSC-conditioned medium. Supplementary Table S1: supplementary table showing the top 100 of up- and downregulated genes in preconditioned MSCs determined by microarray analysis. [file 8888575.f1.zip › Figure S1 (1).pdf]

Figure S1: Inhibition of Nor1 expression and Akt activity in iPS-CM. (**a**) iPS-CM were transfected with Nor1 siRNA and control siRNA, respectively. Knockdown of *Nor1* expression was verified by Real time PCR. n = 3. (**b**) iPS-CM were treated with 400 nM wortmanin and the expression of phosphorylated Akt was proven by Western blot. n = 3. $^{**}p < 0.01$, $^{***}p < 0.001$.

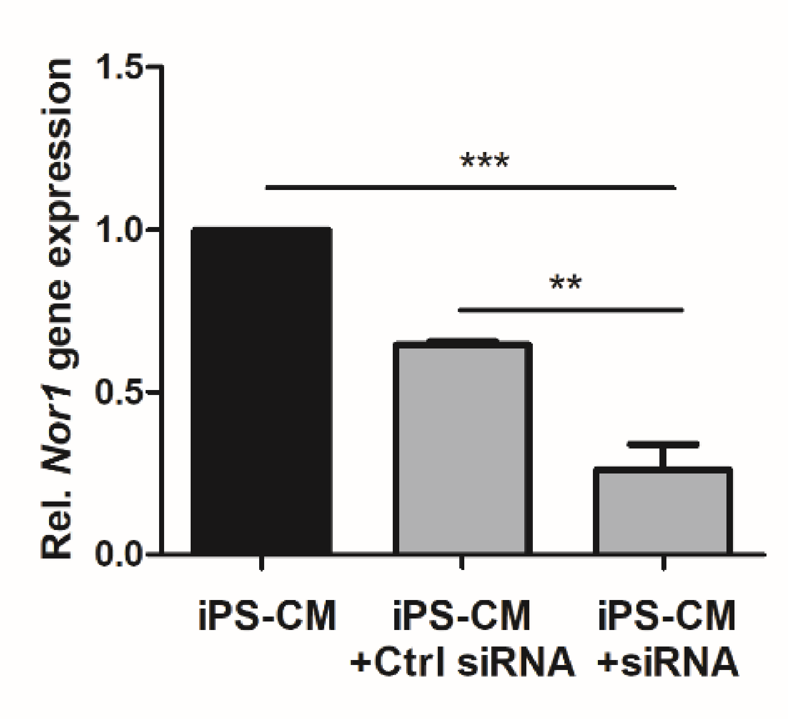

(a)

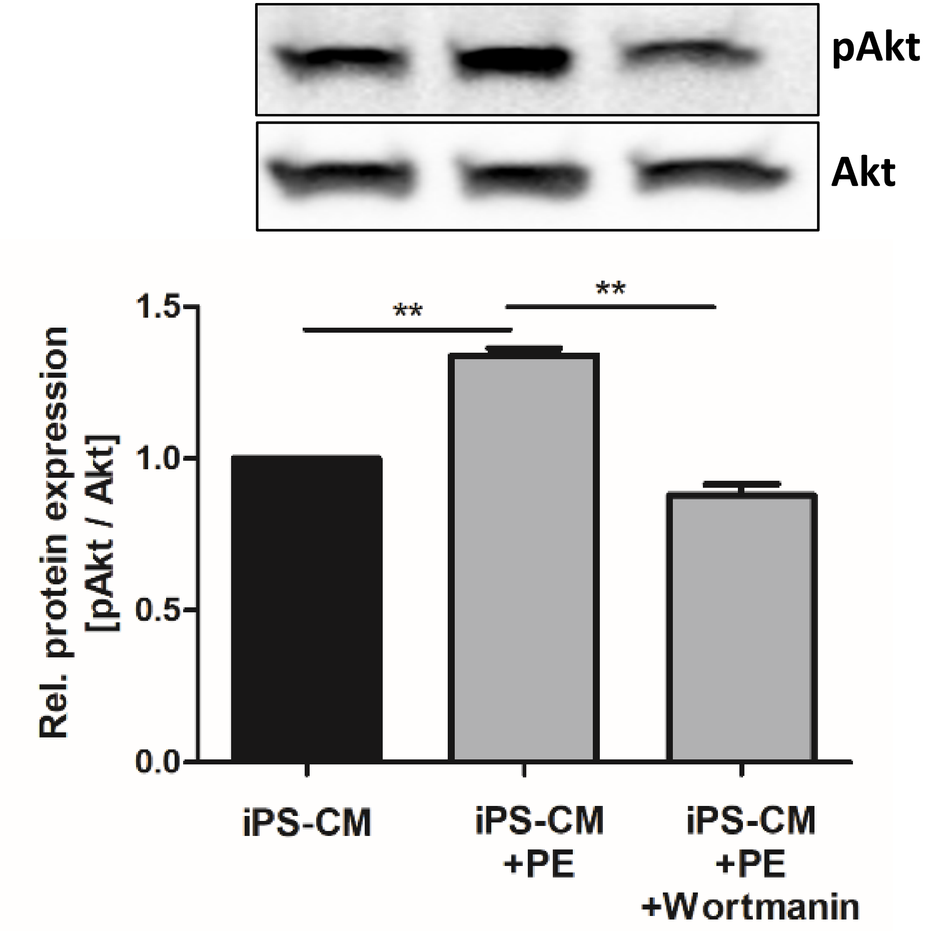

(b)
