# Supplementary material for: VEGF Contributes to Mesenchymal Stem Cell-Mediated Reversion of Nor1-Dependent Hypertrophy in iPS Cell-Derived Cardiomyocytes
Source: Stem Cells Int. 2021 Apr 10;2021:8888575. doi: 10.1155/2021/8888575 (PMC8053052; doi:10.1155/2021/8888575)

Figure S3: Effect of different concentrations of MSC-conditioned medium on hypertrophy regression. PE-treated, hypertrophied iPS-CM were co-cultured with preconditioned MSCs (acMSCs) seeded in the upper chamber of transwell inserts (0.4 µm pore size) or in medium supplemented with 1% - 30% MSC-conditioned medium (CM) for 24 h. Cell area was quantified by F-actin staining with Alexa Fluor 555-conjugated phalloidin. n = 3. *$p < 0.05$, ***$p < 0.001$.

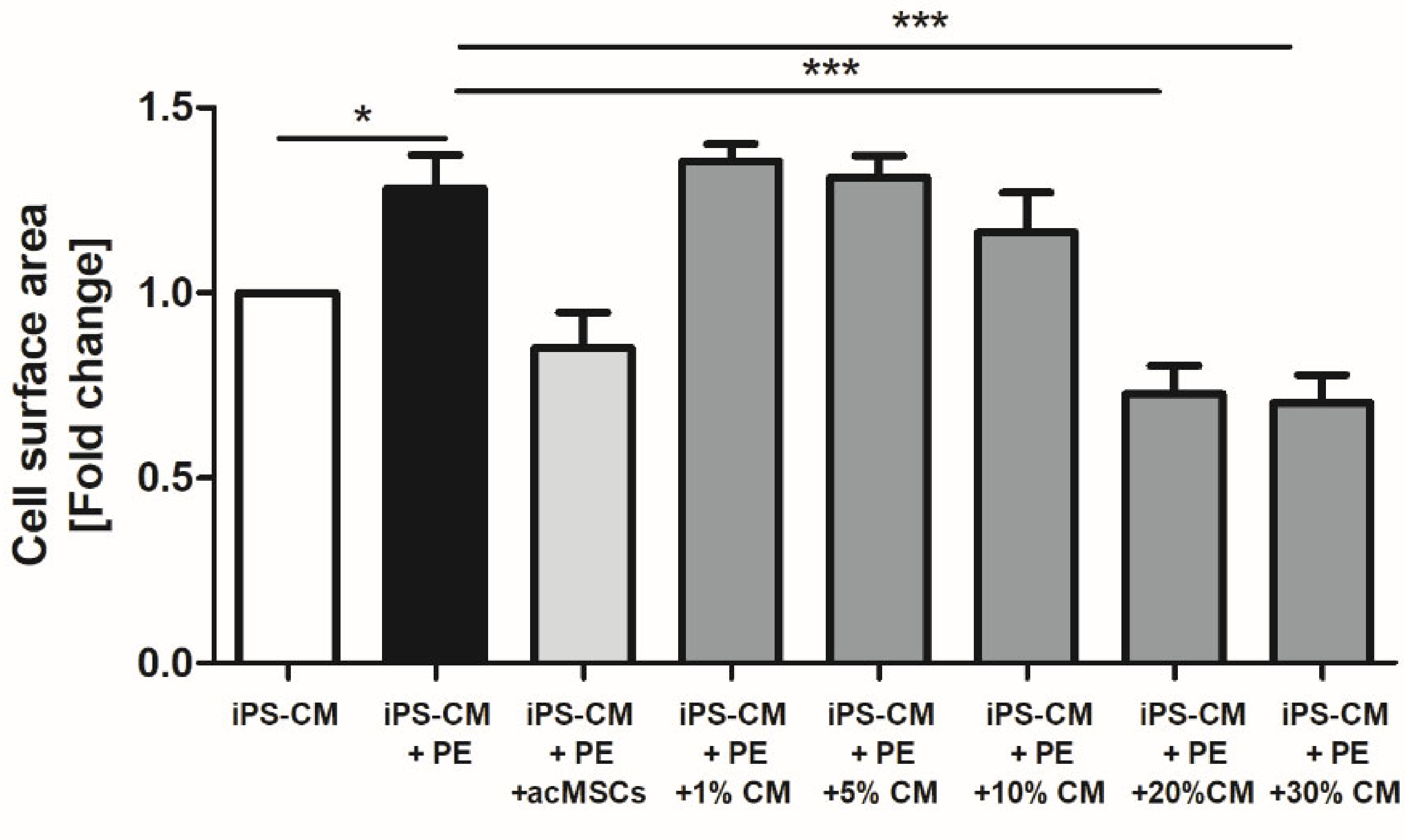

Supplement: Supplementary Materials — Supplementary Figure S1: supplementary figure showing suppression of Nor1 expression and inhibition of Akt activity by siRNA transfection and wortmannin treatment, respectively. Supplementary Figure S2: supplementary figure showing NF-κB activation and HIF-1α upregulation in preconditioned MSCs. Supplementary Figure S3: supplementary figure showing hypertrophy regression in iPS-CM after incubation with different concentrations of MSC-conditioned medium. Supplementary Table S1: supplementary table showing the top 100 of up- and downregulated genes in preconditioned MSCs determined by microarray analysis. [file 8888575.f1.zip › Figure S3 (1).pdf]
