# Supplementary material for: VEGF Contributes to Mesenchymal Stem Cell-Mediated Reversion of Nor1-Dependent Hypertrophy in iPS Cell-Derived Cardiomyocytes
Source: Stem Cells Int. 2021 Apr 10;2021:8888575. doi: 10.1155/2021/8888575 (PMC8053052; doi:10.1155/2021/8888575)
Supplement: Supplementary Materials — Supplementary Figure S1: supplementary figure showing suppression of Nor1 expression and inhibition of Akt activity by siRNA transfection and wortmannin treatment, respectively. Supplementary Figure S2: supplementary figure showing NF-κB activation and HIF-1α upregulation in preconditioned MSCs. Supplementary Figure S3: supplementary figure showing hypertrophy regression in iPS-CM after incubation with different concentrations of MSC-conditioned medium. Supplementary Table S1: supplementary table showing the top 100 of up- and downregulated genes in preconditioned MSCs determined by microarray analysis. [file 8888575.f1.zip › Table S1.docx]

**Table S1**: Top 100 of up- and down-regulated genes in preconditioned MSCs

| Gene symbol | | Gene ID | | Stimulated Avg (log2) | | Control Avg (log2) | | Fold change | | | P value | | | |
| --- | --- | --- | --- | --- | --- | --- | --- | --- | --- | --- | --- | --- | --- | --- |
| Cxcl9 | TC0500002755.mm.2 | | 18,2 | | 4,29 | | 15342,71 | | 8,54E-11 | | |  |  |  |
| Lcn2 | TC0200003303.mm.2 | | 16,86 | | 3,77 | | 8751,09 | | 3,86E-12 | | |  |  |  |
| Ccl3 | TC1100003401.mm.2 | | 15,89 | | 4,04 | | 3678,99 | | 2,08E-10 | | |  |  |  |
| Ccl5 | TC1100003396.mm.2 | | 17,67 | | 5,93 | | 3403,65 | | 3,96E-11 | | |  |  |  |
| Nos2 | TC1100001171.mm.2 | | 16,29 | | 4,59 | | 3318,96 | | 6,48E-11 | | |  |  |  |
| Saa3 | TC0700003039.mm.2 | | 16,67 | | 5,39 | | 2474,94 | | 9,91E-11 | | |  |  |  |
| Gm12250 | TC1100000681.mm.2 | | 16,14 | | 4,93 | | 2365,35 | | 3,88E-11 | | |  |  |  |
| C3 | TC1700002352.mm.2 | | 15,58 | | 4,61 | | 2002,89 | | 1,21E-09 | | |  |  |  |
| Cxcl5 | TC0500000841.mm.2 | | 15,36 | | 4,82 | | 1495,33 | | 1,22E-10 | | | | |  |
| Cxcl10 | TC0500002756.mm.2 | | 17,87 | | 7,51 | | 1312,81 | | 7,47E-10 | | |  |  |  |
| Cxcl11 | TC0500002757.mm.2 | | 15,33 | | 5,88 | | 702,77 | | 3,93E-10 | | |  |  |  |
| Igtp | TC1100004265.mm.2 | | 16 | | 6,65 | | 653,99 | | 5,34E-11 | | |  |  |  |
| Gbp2b; Gbp5 | TC0300001444.mm.2 | | 14,53 | | 5,18 | | 650,68 | | 3,96E-11 | | |  |  |  |
| Mpeg1 | TC1900000246.mm.2 | | 13,43 | | 4,31 | | 553,87 | | 9,07E-11 | | |  |  |  |
| Slc15a3 | TC1900000211.mm.2 | | 13,32 | | 4,39 | | 489,6 | | 2,08E-10 | | |  |  |  |
| Il6 | TC0500000283.mm.2 | | 16,42 | | 7,54 | | 471,33 | | 3,01E-08 | | |  |  |  |
| Iigp1 | TC1800000610.mm.2 | | 16,83 | | 8,05 | | 438,55 | | 4,41E-09 | | |  |  |  |
| Gm4841 | TC1800001396.mm.2 | | 14,62 | | 5,85 | | 435,8 | | 1,83E-10 | | |  |  |  |
| Cd274 | TC1900000441.mm.2 | | 13,57 | | 4,93 | | 397,16 | | 1,67E-10 | | |  |  |  |
| Gm4951 | TC1800000606.mm.2 | | 14,04 | | 5,59 | | 348,69 | | 1,83E-10 | | |  |  |  |
| Apol6 | TC1500000617.mm.2 | | 13,91 | | 5,47 | | 345,49 | | 3,96E-11 | | |  |  |  |
| Ch25h | TC1900001418.mm.2 | | 13,13 | | 4,76 | | 331,68 | | 6,48E-11 | | |  |  |  |
| F830016B08Rik | TC1800000609.mm.2 | | 13,08 | | 4,74 | | 322,41 | | 6,65E-10 | | |  |  |  |
| Fam26f | TC1000001994.mm.2 | | 12,26 | | 3,95 | | 317,4 | | 3,96E-11 | | |  |  |  |
| Gbp4 | | TC0500003731.mm.2 | | 17,03 | | 8,73 | | 316,28 | | 1,09E-09 | |  |  |  |
| Batf2 | | TC1900000102.mm.2 | | 13,24 | | 5,08 | | 285,15 | | 2,08E-10 | |  |  |  |
| Gbp8 | | TC0500003729.mm.2 | | 13,9 | | 5,77 | | 280,71 | | 5,89E-10 | |  |  |  |
| Psmb9 | | TC1700001902.mm.2 | | 14,52 | | 6,69 | | 227,42 | | 3,34E-10 | | |  |  |
| H2-Q4 | | TC1700000684.mm.2 | | 13,56 | | 5,78 | | 220,58 | | 1,29E-09 | | |  |  |
| Gbp10; Gbp6 | | TC0500002895.mm.2 | | 17,56 | | 9,94 | | 197,16 | | 2,75E-09 | | |  |  |
| AA467197; Mir147 | | TC0200001854.mm.2 | | 14,68 | | 7,11 | | 189,88 | | 4,38E-09 | | |  |  |
| Serping1 | | TC0200003880.mm.2 | | 16,34 | | 8,8 | | 186,38 | | 1,75E-09 | | |  |  |
| Trim30a | | TC0700003943.mm.2 | | 13,8 | | 6,28 | | 183,26 | | 1,03E-08 | | |  |  |
| Cxcl1 | | TC0500000848.mm.2 | | 14,38 | | 6,89 | | 180,25 | | 1,54E-10 | | |  |  |
| Cd40 | | TC0200002544.mm.2 | | 12,56 | | 5,25 | | 159,63 | | 6,78E-10 | | |  |  |
| Pydc3 | | TC0100001634.mm.2 | | 11,94 | | 4,64 | | 157,25 | | 5,89E-10 | | |  |  |
| Socs1 | | TC1600001198.mm.2 | | 12,7 | | 5,55 | | 142,07 | | 1,67E-10 | | |  |  |
| Phf11b | | TC1400002162.mm.2 | | 13,12 | | 6,07 | | 131,96 | | 3,34E-10 | | |  |  |
| Gbp3 | | TC0300001446.mm.2 | | 16,64 | | 9,69 | | 124,24 | | 6,37E-10 | | |  |  |
| Cmpk2 | | TC1200000225.mm.2 | | 12,68 | | 5,82 | | 115,71 | | 1,74E-09 | | |  |  |
| Gbp11 | | TC0500002896.mm.2 | | 14,65 | | 7,89 | | 108,36 | | 1,67E-10 | | |  |  |
| Ccrl2 | | TC0900003125.mm.2 | | 10,8 | | 4,11 | | 103,28 | | 1,83E-10 | | |  |  |
| Irf7 | | TC0700004530.mm.2 | | 15,74 | | 9,07 | | 101,89 | | | 2,00E-09 | | | |
| Zbp1 | | TC0200005290.mm.2 | | 13,2 | | 6,63 | | 95,4 | | | 1,47E-09 | | | |
| Lif | | TC1100000041.mm.2 | | 11,56 | | 5,17 | | 84,39 | | | 1,92E-07 | | | |
| Hba-a2; Hba-a1 | | TC1100000336.mm.2 | | 11,06 | | 4,66 | | 84,29 | | | 2,12E-07 | | | |
| Hba-a1; Hba-a2 | | TC1100000339.mm.2 | | 11,06 | | 4,67 | | 84,03 | | | 2,12E-07 | | | |
| Psmb8 | | TC1700000622.mm.2 | | 15,33 | | 9,08 | | 76,34 | | | 8,60E-10 | | | |
| Ifit1 | | TC1900000504.mm.2 | | 15,1 | | 8,87 | | 75,16 | | | 2,12E-08 | | | |
| Upp1 | | TC1100000119.mm.2 | | 10,27 | | 4,15 | | 69,72 | | | 5,26E-07 | | | |
| Serpina3f | | TC1200002524.mm.2 | | 14,23 | | 8,11 | | 69,62 | | | 4,18E-09 | | | |
| Il18bp | | TC0700003850.mm.2 | | 13,14 | | 7,06 | | 67,53 | | | 2,61E-09 | | | |
| Ifit3b | | TC1900000502.mm.2 | | 17,37 | | 11,31 | | 66,69 | | | 5,89E-10 | | | |
| Nlrc5; Mir7072 | | TC0800001110.mm.2 | | 10,28 | | 4,27 | | 64,53 | | | 1,85E-08 | | | |
| Ripk2 | | TC0400002251.mm.2 | | 14,46 | | 8,46 | | 63,97 | | | 3,32E-10 | | | |
| Stc2 | | TC1100002457.mm.2 | | 12,27 | | 6,28 | | 63,71 | | | 8,60E-10 | | | |
| Ccl7 | | TC1100001220.mm.2 | | 16,59 | | 10,61 | | 63,21 | | | 8,60E-10 | | | |
| Irgm2 | | TC1100004266.mm.2 | | 14,62 | | 8,68 | | 61,25 | | | 5,00E-09 | | | |
| Tap1 | | TC1700000621.mm.2 | | 16,28 | | 10,35 | | 61,11 | | | 1,60E-08 | | | |
| Oas1a | | TC0500003181.mm.2 | | 9,97 | | 4,04 | | 60,69 | | | 2,34E-08 | | | |
| Wfdc17 | | TC1100001264.mm.2 | | 10,85 | | 4,95 | | 59,9 | | | 2,00E-08 | | | |
| Ifit3 | | TC1900000501.mm.2 | | 17,37 | | 11,51 | | 57,98 | | | 2,08E-10 | | | |
| Cxcl16 | | TC1100003097.mm.2 | | 15,16 | | 9,33 | | 57 | | | 5,61E-10 | | | |
| Htr2a | | TC1400001045.mm.2 | | 12,21 | | 6,46 | | 54,07 | | | 4,25E-08 | | | |
| Gm18853 | | TC0700004003.mm.2 | | 12,78 | | 7,08 | | 51,99 | | | 4,57E-08 | | | |
| Apol9a | | TC1500001809.mm.2 | | 13,4 | | 7,71 | | 51,62 | | | 2,06E-08 | | | |
| Gbp2 | | TC0300001447.mm.2 | | 17,61 | | 11,93 | | 51,43 | | | 5,38E-10 | | | |
| Gm4070; Gvin1; Gm17757; Gm18853 | | TC0700003996.mm.2 | | 12,51 | | 6,83 | | 51,23 | | | 4,50E-08 | | | |
| Ly6c2 | | TC1500001730.mm.2 | | 12,43 | | 6,78 | | 50,27 | | | 6,89E-07 | | | |
| Mx2 | | TC1600001092.mm.2 | | 13,08 | | 7,49 | | 48,29 | | | 5,89E-10 | | | |
| Ly6c1 | | TC1500001729.mm.2 | | 16,39 | | 10,8 | | 48,23 | | | 2,74E-08 | | | |
| Ms4a4d | | TC1900000226.mm.2 | | 15,21 | | 9,64 | | 47,39 | | | 5,76E-08 | | | |
| Slfn5 | | TC1100001236.mm.2 | | 11,09 | | 5,56 | | 46,31 | | | 1,11E-07 | | | |
| Trim30c | | TC0700003942.mm.2 | | 9,06 | | 3,53 | | 46,24 | | | 1,60E-08 | | | |
| Ndufa4l2 | | TC1000001566.mm.2 | | 11,27 | | 5,76 | | 45,7 | | | 4,01E-06 | | | |
| Ccl2 | | TC1100001219.mm.2 | | 17,82 | | 12,32 | | 45,28 | | | 1,75E-09 | | | |
| Gbp7 | | TC0300001445.mm.2 | | 16,3 | | 10,88 | | 43,03 | | | 5,00E-09 | | | |
| Phf11d; Phf11c | | TC1400002869.mm.2 | | 15,15 | | 9,77 | | 41,67 | | | 3,82E-09 | | | |
| Pla1a | | TC1600001604.mm.2 | | 12,66 | | 7,29 | | 41,36 | | | 5,89E-10 | | | |
| Parp14 | | TC1600001556.mm.2 | | 12,93 | | 7,57 | | 41,3 | | | 4,41E-09 | | | |
| Steap4 | | TC0500000043.mm.2 | | 10,22 | | 4,86 | | 41 | | | 3,66E-09 | | | |
| Gm8979; Gm8989 | | TC0700003998.mm.2 | | 11,7 | | 6,36 | | 40,57 | | | 1,03E-08 | | | |
| Gm8989 | | TC0700004007.mm.2 | | 11,7 | | 6,36 | | 40,57 | | | 1,03E-08 | | | |
| H2-L; H2-D1 | | TC1700002790.mm.2 | | 15,12 | | 9,79 | | 40,16 | | | 6,84E-09 | | | |
| Oasl2 | | TC0500001239.mm.2 | | 16,12 | | 10,81 | | 39,67 | | | 2,54E-09 | | | |
| Gvin1 | | TC0700004002.mm.2 | | 15,55 | | 10,25 | | 39,4 | | | 1,47E-09 | | | |
| Tgtp2 | | TC1100002649.mm.2 | | 17,41 | | 12,17 | | 37,73 | | | 5,00E-09 | | | |
| Ifitm1 | | TC0700002034.mm.2 | | 15,62 | | 10,38 | | 37,71 | | | 2,63E-08 | | | |
| Ifi47; Olfr56 | | TC1100000496.mm.2 | | 14,33 | | 9,13 | | 36,68 | | | 1,29E-07 | | | |
| Herc6 | | TC0600003503.mm.2 | | 13,9 | | 8,74 | | 35,92 | | | 5,98E-09 | | | |
| Ifi44l | | TC0300003134.mm.2 | | 9,52 | | 4,36 | | 35,86 | | | 1,07E-08 | | | |
| Usp18 | | TC0600001369.mm.2 | | 14,47 | | 9,34 | | 35,09 | | | 6,53E-09 | | | |
| Slamf8 | | TC0100003575.mm.2 | | 8,8 | | 3,67 | | 35,08 | | | 1,92E-07 | | | |
| Hk2 | | TC0600002550.mm.2 | | 12,95 | | 7,82 | | 35,07 | | | 2,75E-08 | | | |
| Xdh | | TC1700002526.mm.2 | | 15,52 | | 10,4 | | 34,81 | | | 1,03E-08 | | | |
| C2 | | TC1700002817.mm.2 | | 10,55 | | 5,45 | | 34,15 | | | 1,77E-08 | | | |
| Rsad2 | | TC1200001552.mm.2 | | 11,43 | | 6,37 | | 33,41 | | | 1,66E-08 | | | |
| Rnd1 | | TC1500002183.mm.2 | | 12,37 | | 7,37 | | 32,01 | | | 4,50E-08 | | | |
| Pim1 | | TC1700000505.mm.2 | | 13,28 | | 8,32 | | 31,17 | | | 1,75E-08 | | | |
| Trim21 | | TC0700003862.mm.2 | | 12,12 | | 7,2 | | 30,15 | | | 2,18E-08 | | | |
| Rab3il1 | | TC1900000184.mm.2 | | 6,43 | | 9,49 | | -8,35 | | | 3,64E-05 | | | |
| Sema5a | | TC1500000217.mm.2 | | 8,02 | | 11,12 | | -8,56 | | | 2,81E-07 | | | |
| Spns2 | | TC1100003146.mm.2 | | 3,96 | | 7,07 | | -8,59 | | | 0,002 | | | |
| Medag | | TC0500003722.mm.2 | | 11,58 | | 14,69 | | -8,6 | | | 1,04E-06 | | | |
| Mgp | | TC0600003335.mm.2 | | 8,8 | | 11,91 | | -8,61 | | | 1,41E-05 | | | |
| Ptn | | TC0600002079.mm.2 | | 7,69 | | 10,81 | | -8,67 | | | 6,80E-06 | | | |
| Col3a1 | | TC0100000343.mm.2 | | 13,07 | | 16,23 | | -8,89 | | | 3,92E-06 | | | |
| Fkbp7 | | TC0200003806.mm.2 | | 10,72 | | 13,88 | | -8,94 | | | 1,11E-06 | | | |
| Apcdd1 | | TC1800000646.mm.2 | | 4,92 | | 8,08 | | -8,96 | | | 2,16E-06 | | | |
| Gas6 | | TC0800001737.mm.2 | | 6,99 | | 10,16 | | -8,99 | | | 2,77E-06 | | | |
| Maged2 | | TC0X00003168.mm.2 | | 9,48 | | 12,65 | | -9,01 | | | 8,45E-07 | | | |
| Gamt | | TC1000002493.mm.2 | | 8,34 | | 11,53 | | -9,1 | | | 3,12E-06 | | | |
| Mid1 | | TC0Y00000221.mm.2 | | 12,35 | | 15,54 | | -9,12 | | | 4,16E-07 | | | |
| Mid1 | | TC0Y00000224.mm.2 | | 12,35 | | 15,54 | | -9,12 | | | 4,16E-07 | | | |
| Srpx2 | | TC0X00001271.mm.2 | | 12 | | 15,19 | | -9,16 | | | 1,11E-07 | | | |
| Cav1 | | TC0600000128.mm.2 | | 7,81 | | 11,01 | | -9,19 | | | 6,60E-07 | | | |
| Kirrel3 | | TC0900000313.mm.2 | | 6,99 | | 10,21 | | -9,31 | | | 7,93E-08 | | | |
| Jag1 | | TC0200004713.mm.2 | | 7,31 | | 10,55 | | -9,4 | | | 8,69E-07 | | | |
| Gxylt2 | | TC0600001154.mm.2 | | 8,6 | | 11,83 | | -9,4 | | | 8,05E-07 | | | |
| Mir322; Mir351; Mir503; C430049B03Rik | | TC0X00002234.mm.2 | | 5,47 | | 8,73 | | -9,55 | | | 2,81E-07 | | | |
| Jup | | TC1100003769.mm.2 | | 8,03 | | 11,31 | | -9,72 | | | 3,42E-08 | | | |
| Enpp3 | | TC1000001895.mm.2 | | 6,43 | | 9,73 | | -9,86 | | | 2,49E-06 | | | |
| Igsf10 | | TC0300002056.mm.2 | | 6,42 | | 9,72 | | -9,87 | | | 5,56E-06 | | | |
| Prrx2 | | TC0200000543.mm.2 | | 8,03 | | 11,37 | | -10,12 | | | 4,50E-08 | | | |
| Dpep1 | | TC0800001511.mm.2 | | 6,16 | | 9,52 | | -10,25 | | | 7,00E-07 | | | |
| Pde5a | | TC0300001249.mm.2 | | 8,49 | | 11,88 | | -10,48 | | | 2,40E-07 | | | |
| Tead2 | | TC0700000835.mm.2 | | 6,7 | | 10,1 | | -10,56 | | | 1,19E-07 | | | |
| Synpo | | TC1800001402.mm.2 | | 9,74 | | 13,14 | | -10,56 | | | 7,39E-08 | | | |
| Sdc2 | | TC1500000221.mm.2 | | 12,1 | | 15,5 | | -10,57 | | | 8,60E-07 | | | |
| Serpine2 | | TC0100002645.mm.2 | | 8,72 | | 12,12 | | -10,59 | | | 0,0017 | | | |
| Frzb | | TC0200003834.mm.2 | | 6,72 | | 10,12 | | -10,62 | | | 1,49E-06 | | | |
| Hcn1 | | TC1300001330.mm.2 | | 4,38 | | 7,81 | | -10,82 | | | 4,01E-07 | | | |
| Mir675; H19 | | TC0700004569.mm.2 | | 4,74 | | 8,19 | | -10,93 | | | 8,45E-07 | | | |
| Smoc2 | | TC1700000168.mm.2 | | 8,34 | | 11,8 | | -10,97 | | | 1,32E-07 | | | |
| Prss23 | | TC0700003714.mm.2 | | 11,33 | | 14,81 | | -11,15 | | | 2,43E-06 | | | |
| Col6a1 | | TC1000002406.mm.2 | | 11,76 | | 15,28 | | -11,43 | | | 7,72E-07 | | | |
| Epha7 | | TC0400000234.mm.2 | | 9,72 | | 13,25 | | -11,51 | | | 3,52E-08 | | | |
| Crabp1 | | TC0900000662.mm.2 | | 8,5 | | 12,02 | | -11,52 | | | 4,14E-07 | | | |
| Gsta4 | | TC0900001048.mm.2 | | 10,03 | | 13,57 | | -11,6 | | | 6,38E-07 | | | |
| Palm2; Pakap | | TC0400004176.mm.2 | | 7,45 | | 11,01 | | -11,73 | | | 2,19E-05 | | | |
| Sned1; Mir6901 | | TC0100000927.mm.2 | | 6,67 | | 10,24 | | -11,83 | | | 3,00E-07 | | | |
| Mid1 | | TC0X00001710.mm.2 | | 11,18 | | 14,76 | | -11,89 | | | 1,92E-07 | | | |
| Slc9a9 | | TC0900001182.mm.2 | | 7,45 | | 11,03 | | -11,93 | | | 1,65E-07 | | | |
| Irs1 | | TC0100002667.mm.2 | | 7,49 | | 11,15 | | -12,66 | | | 1,07E-06 | | | |
| Tgfb2 | | TC0100003754.mm.2 | | 9,01 | | 12,68 | | -12,68 | | | 3,32E-06 | | | |
| Fam101b | | TC1100003236.mm.2 | | 5,85 | | 9,54 | | -12,91 | | | 1,13E-07 | | | |
| Clec11a | | TC0700002957.mm.2 | | 6,48 | | 10,18 | | -13,05 | | | 1,05E-07 | | | |
| Pi15 | | TC0100000112.mm.2 | | 4,21 | | 7,91 | | -13,06 | | | 5,02E-07 | | | |
| Dpt | | TC0100001510.mm.2 | | 9,47 | | 13,21 | | -13,35 | | | 1,86E-05 | | | |
| Matn2 | | TC1500000237.mm.2 | | 9,68 | | 13,47 | | -13,89 | | | 3,49E-07 | | | |
| Chrdl1 | | TC0X00003113.mm.2 | | 8,21 | | 12,02 | | -14 | | | 1,22E-07 | | | |
| Tox | | TC0400002159.mm.2 | | 5,51 | | 9,32 | | -14,02 | | | 8,02E-07 | | | |
| Bnc2 | | TC0400002962.mm.2 | | 8,2 | | 12,04 | | -14,28 | | | 1,50E-07 | | | |
| Emilin1 | | TC0500000305.mm.2 | | 7,75 | | 11,6 | | -14,38 | | | 2,26E-06 | | | |
| Gas1 | | TC1300002131.mm.2 | | 6,99 | | 10,91 | | -15,16 | | | 7,73E-08 | | | |
| Thsd7a | | TC0600001867.mm.2 | | 8,53 | | 12,45 | | -15,18 | | | 6,15E-07 | | | |
| Ncam1 | | TC0900002254.mm.2 | | 8,72 | | 12,67 | | -15,46 | | | 8,10E-09 | | | |
| Glt8d2 | | TC1000002571.mm.2 | | 8,05 | | 12,07 | | -16,22 | | | 3,58E-08 | | | |
| Sema3d | | TC0500000080.mm.2 | | 6,1 | | 10,15 | | -16,53 | | | 1,46E-07 | | | |
| Tnmd | | TC0X00001270.mm.2 | | 5,68 | | 9,74 | | -16,69 | | | 8,17E-07 | | | |
| Hpgd | | TC0800000621.mm.2 | | 4,53 | | 8,74 | | -18,48 | | | 4,71E-06 | | | |
| Itm2a | | TC0X00002845.mm.2 | | 6,35 | | 10,64 | | -19,55 | | | 1,47E-05 | | | |
| Lmo7 | | TC1400001177.mm.2 | | 12,31 | | 16,66 | | -20,43 | | | 3,43E-08 | | | |
| Wisp2 | | TC0200002496.mm.2 | | 8,46 | | 12,9 | | -21,67 | | | 1,28E-06 | | | |
| Col6a2 | | TC1000002405.mm.2 | | 9,8 | | 14,31 | | -22,84 | | | 5,67E-08 | | | |
| Fgl2 | | TC0500000171.mm.2 | | 9,22 | | 13,73 | | -22,87 | | | 3,58E-08 | | | |
| Adamts1 | | TC1600001974.mm.2 | | 10,07 | | 14,58 | | -22,87 | | | 1,35E-08 | | | |
| Sorbs2 | | TC0800000516.mm.2 | | 7,3 | | 11,86 | | -23,47 | | | 0,0001 | | | |
| Nid2 | | TC1400000158.mm.2 | | 6,2 | | 10,78 | | -23,87 | | | 1,26E-08 | | | |
| Prelp | | TC0100003099.mm.2 | | 8,19 | | 12,84 | | -25,15 | | | 4,83E-08 | | | |
| Itgbl1 | | TC1400001346.mm.2 | | 8,4 | | 13,08 | | -25,54 | | | 3,76E-09 | | | |
| Peg10 | | TC0600000014.mm.2 | | 9,64 | | 14,32 | | -25,69 | | | 2,08E-06 | | | |
| Vsnl1 | | TC1200001421.mm.2 | | 10,42 | | 15,2 | | -27,4 | | | 5,02E-07 | | | |
| H2-M1 | | TC1700002031.mm.2 | | 3,45 | | 8,26 | | -28,09 | | | 2,40E-06 | | | |
| Myl9 | | TC0200002399.mm.2 | | 7,7 | | 12,51 | | -28,11 | | | 3,52E-08 | | | |
| Bmp4 | | TC1400001914.mm.2 | | 4,22 | | 9,05 | | -28,42 | | | 7,39E-06 | | | |
| Col11a1 | | TC0300001173.mm.2 | | 5,31 | | 10,19 | | -29,5 | | | 4,23E-08 | | | |
| Creb3l1 | | TC0200004133.mm.2 | | 6,72 | | 11,62 | | -29,81 | | | 1,44E-05 | | | |
| Gulp1 | | TC0100000338.mm.2 | | 9,72 | | 14,63 | | -30,16 | | | 4,95E-08 | | | |
| Zfp521 | | TC1800001005.mm.2 | | 5,84 | | 10,79 | | -30,93 | | | 3,52E-08 | | | |
| Gas2 | | TC0700000917.mm.2 | | 6,79 | | 11,82 | | -32,58 | | | 2,42E-08 | | | |
| Osr1 | | TC1200000083.mm.2 | | 8,46 | | 13,49 | | -32,67 | | | 2,63E-08 | | | |
| Igf1 | | TC1000001066.mm.2 | | 6,82 | | 11,87 | | -33,11 | | | 2,37E-08 | | | |
| Adamts5 | | TC1600001976.mm.2 | | 7,79 | | 12,92 | | -34,85 | | | 1,03E-08 | | | |
| Pik3r3 | | TC0400001250.mm.2 | | 8,31 | | 13,48 | | -35,97 | | | 1,55E-08 | | | |
| Cnn1 | | TC0900000212.mm.2 | | 6,2 | | 11,39 | | -36,54 | | | 2,12E-07 | | | |
| Itga11 | | TC0900000791.mm.2 | | 6,68 | | 12,05 | | -41,36 | | | 6,29E-08 | | | |
| Postn | | TC0300000408.mm.2 | | 7,69 | | 13,16 | | -44,38 | | | 8,06E-07 | | | |
| Rgs4 | | TC0100003502.mm.2 | | 5,32 | | 11,15 | | -56,64 | | | 1,40E-06 | | | |
| Sfrp2 | | TC0300000647.mm.2 | | 6,51 | | 12,58 | | -67,26 | | | 2,63E-08 | | | |
| Mfap2 | | TC0400001774.mm.2 | | 4,07 | | 10,2 | | -69,83 | | | 5,89E-10 | | | |
| Ccdc3 | | TC0200000048.mm.2 | | 5 | | 11,15 | | -70,96 | | | 2,10E-07 | | | |
| Ogn | | TC1300000569.mm.2 | | 11,82 | | 18,14 | | -80,14 | | | 2,54E-07 | | | |
| Aoc3 | | TC1100001651.mm.2 | | 4,25 | | 10,61 | | -82,33 | | | 1,63E-08 | | | |
| Fmod | | TC0100001218.mm.2 | | 4,77 | | 11,35 | | -95,31 | | | 2,36E-08 | | | |
| Mest | | TC0600000277.mm.2 | | 4,41 | | 11,49 | | -135,55 | | | 2,05E-07 | | | |
| Rgs2 | | TC0100003204.mm.2 | | 4,9 | | 12,03 | | -140,63 | | | 1,83E-10 | | | |
| Penk | | TC0400002139.mm.2 | | 6,87 | | 14,3 | | -172,74 | | | 1,85E-08 | | | |
| Omd | | TC1300000568.mm.2 | | 5,38 | | 12,84 | | -175,41 | | | 6,53E-09 | | | |
| Actg2 | | TC0600002574.mm.2 | | 4,39 | | 11,86 | | -177,68 | | | 1,47E-05 | | | |
| Lrrc17 | | TC0500000173.mm.2 | | 5,13 | | 12,82 | | -206,19 | | | 1,59E-10 | | | |
| Adh1 | | TC0300001409.mm.2 | | 4,68 | | 13,36 | | -408,8 | | | 1,22E-10 | | | |
